# An industrialized diet as a determinant of methylation in the 1F region of the *NR3C1* gene promoter

**DOI:** 10.3389/fnut.2024.1168715

**Published:** 2024-04-03

**Authors:** Tamires dos Santos Vieira, Flávia Vitorino Freitas, Luiz Cláudio Barreto Silva Neto, Aline Ribeiro Borçoi, Suzanny Oliveira Mendes, Amanda Sgrancio Olinda, Ivana Alece Arantes Moreno, Bárbara Risse Quaioto, Marcele Lorentz Mattos de Souza, Wagner Miranda Barbosa, Juliana Krüger Arpini, Bruna Pereira Sorroche, Julia de Assis Pinheiro, Anderson Barros Archanjo, Joaquim Gasparini dos Santos, Lidia Maria Rebolho Batista Arantes, Daniela Rodrigues de Oliveira, Adriana Madeira Alvares da Silva

**Affiliations:** ^1^Program of Post-Graduation in Biotechnology/Renorbio, Federal University of Espírito Santo, Vitória, Brazil; ^2^Department of Pharmacy and Nutrition, Federal University of Espírito Santo, Alegre, Brazil; ^3^Program of Post-Graduation in Nutrition and Health, Federal University of Espírito Santo, Vitória, Brazil; ^4^Department of Morphology, Federal University of Espirito Santo, Vitória, Brazil; ^5^Department of Biology, Federal University of Espirito Santo, Alegre, Brazil; ^6^Molecular Oncology Research Center, Barretos Cancer Hospital, Barretos, Brazil; ^7^Department of Pharmacology and Medical Toxicology, College of Wisconsin, Milwaukee, WI, United States

**Keywords:** NR3C1 DNA methylation, food consumption, lifestyle, metabolic stress, chronic diseases, diet’s composition, epigenetic mechanisms, industrialized diet

## Abstract

**Background:**

Dietary composition can modify gene expression, favoring the development of chronic diseases via epigenetic mechanisms.

**Objective:**

Our study aimed to investigate the relationship between dietary patterns and *NR3C1* gene methylation in users of the Brazilian Public Unified Health System (SUS).

**Methods:**

We recruited 250 adult volunteers and evaluated their socioeconomic status, psychosocial characteristics, lifestyle, and anthropometrics. Peripheral blood was collected and evaluated for cortisol levels, glycemia, lipid profile, and insulin resistance; methylation of CpGs 40–47 of the 1F region of the *NR3C1* gene was also measured. Factors associated with degree of methylation were evaluated using generalized linear models (*p* < 0.05). Lifestyle variables and health variables were included as confounding factors.

**Results:**

The findings of our cross-sectional study indicated an association between *NR3C1* DNA methylation and intake of processed foods. We also observed relevant associations of average *NR3C1* DNA across the segment analyzed, methylation in component 1 (40–43), and methylation in component 2 (44–47) with a pattern of consumption of industrialized products in relation to BMI, serum cortisol levels, and lipid profile. These results may indicate a relationship between methylation and metabolic changes related to the stress response.

**Conclusion:**

These findings suggest an association of methylation and metabolic alterations with stress response. In addition, the present study highlights the significant role of diet quality as a stress-inducing factor that influences *NR3C1* methylation. This relationship is further linked to changes in psychosocial factors, lifestyle choices, and cardiometabolic variables, including glucose levels, insulin resistance, and hyperlipidemia.

## 1 Introduction

Worldwide, a process of nutritional, demographic, and epidemiological transition has been observed, and this has been seen to be related to changes in lifestyle: specifically, more sedentary lifestyles and increased adherence to industrialized consumption patterns and calorie-dense diets. These factors have contributed to an increase in chronic noncommunicable diseases (CNCDs) (1). Following this global trend, the Brazilian diet has undergone a reduction in the consumption of natural foods and an increase in the consumption of processed and ultra-processed foods (2).

CNCDs, such as cardiovascular diseases, cancer, diabetes, and chronic respiratory diseases, among others, are responsible for a large percentage of deaths in the world (approximately 74%), and diet and lifestyle are associated with their onset and development (3, 4). Dietary patterns considered healthy and rich in fruits and vegetables are associated with a lower risk of obesity and overweight and, consequently, of CNCDs (5).

Dietary factors are associated with metabolic and molecular alterations that can affect epigenetic phenomena such as DNA methylation, thereby modifying gene expression (6, 7). DNA methylation is a widely researched epigenetic modification involving chemical modification of cytosine, primarily occurring before a guanine residue. When these methylated cytosines are clustered in regions known as “CpG islands” within gene promoters (8), they play a key role in stabilizing and modulating changes in gene expression (9).

It has been reported that an individual’s diet can modify gene expression through epigenetic mechanisms, and such modifications may be associated with the development of CNCDs (7). In that regard, food can be considered as environmental stress, such as pollutants (10–12). The “metabolic stress” related to the consumption of high levels of sugar and fat is gaining importance as a key element that contributes to molecular and epigenetic changes (10–12). Given the increased preference for processed and ultra-processed foods to the detriment of natural foods, investigations on this topic are of great importance (13–15).

Stress comprises an organism’s response to certain sudden or threatening stimuli, resulting in a cascade of signals and feedback loops, in a set of survival responses known as the “fight or flight reaction.” The hypothalamus–pituitary–adrenal (HPA) axis controls the response to environmental stressors, initiated by the release of corticotropin-releasing factor (CRF) secreted by the hypothalamus, which induces the anterior pituitary to secrete adrenocorticotropic hormone (ACTH) into the blood, resulting in the production and release of glucocorticoids by the adrenal cortex (16).

The Nuclear Receptor Subfamily 3-group C-member 1 (*NR3C1*) gene encodes the glucocorticoid receptor (GR), which interacts with cortisol to regulate the functioning of the HPA axis via negative feedback to inhibit cortisol release (17). Methylation of this gene in its promoter region is related to the reduction of GR expression and to dysregulation of the HPA, and is associated with the development of psychiatric disorders in individuals who have suffered psychosocial stress, early trauma, post-traumatic stress, gestational hunger, neglect, and other forms of stress (16, 18–23).

Additionally, some authors have related *NR3C1* methylation to food consumption, considering it to be an important metabolic stress factor (10–12). However, studies on food consumption and epigenetic mechanisms are limited, with a focus on a small number of nutrients and few experimental studies having been conducted (24–26). In this context, it was hypothesized in the present study that greater consumption of processed and ultra-processed foods promotes methylation of the 1F region of the *NR3C1* gene promoter.

In light of the above background, the overall aim of the present study was to evaluate the relationship between *NR3C1* gene methylation levels and patterns of dietary consumption.

## 2 Materials and methods

**Figure 1:**
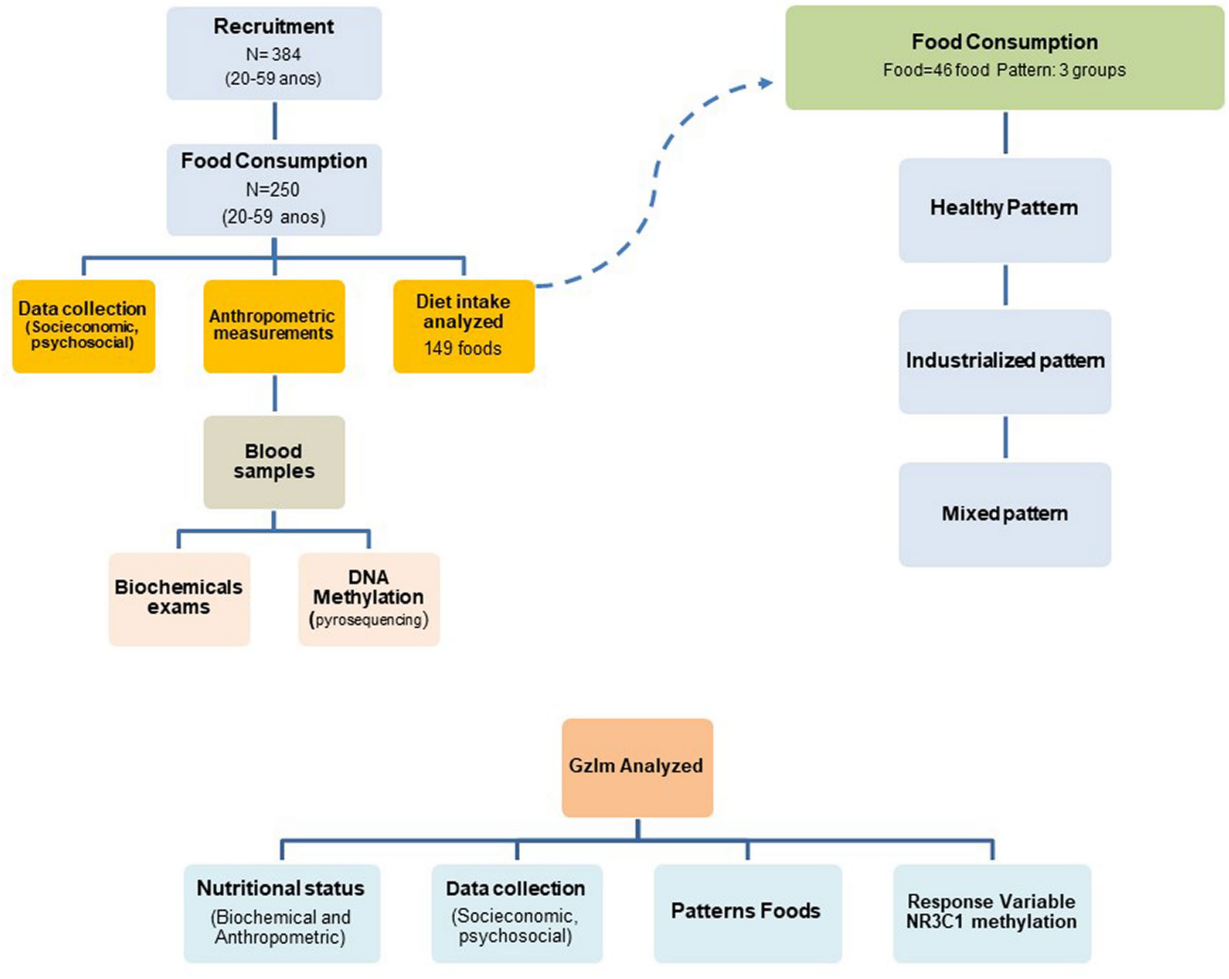
Definition of the sample included in the study.

A total of 384 subjects were recruited at health posts in the city of Alegre, Espirito Santo, Brazil, and with the help of community health workers and volunteers. An investigation into health, lifestyle, food consumption, and epigenetics was carried out. For inclusion in the study, the criteria were: falling into the age group between 20 and 59 years old; not being pregnant; and not presenting with cognitive impairment. Participants who did not meet the inclusion criteria, who were using glucocorticoid medication, for whom complete anthropometric and dietary data were not available, or from whom sufficient biological material for pyrosequencing analysis was not obtained were excluded. Two hundred and fifty individuals met all the inclusion criteria for the study and were included in the final analysis, as illustrated in Figure 1.

All individuals who met the criteria to be included consented to participate by signing the Free and Informed Consent Form (TCLE) relating to their participation in all stages of the study. The study was conducted as approved by the Research Ethics Committee of the Health Sciences Center of the Federal University of Espírito Santo (CEP/CCS/UFES), Vitória, ES, Brazil (Number: 1.574.160 – CAAE: 52830216.5.0000.5060) on 6th June, 2016, and after obtaining a substantiated opinion (number: 3.420.734 – CAAE:08454919.5.0000.8151) on 27th June, 2019.

### 2.1 Assessment of food intake

Dietary intake was assessed using a semi-quantitative food frequency questionnaire (FFQ), adapted from (27) to cover food consumption in the last 6 months. The questionnaire consisted of 127 food items and was administered by trained interviewers and nutritionists to ensure accuracy.

The generated food database was carefully audited. As part of this, each participant’s usual weekly frequency of consumption of each food was transformed into a daily frequency, with 0 representing foods not consumed and 7 representing foods consumed up to 7 times per week (28). The daily frequency of consumption was then calculated by dividing the weekly frequency of consumption by the number of days per week (7 days). The following scores were then given: 0=<=”” *p*=””>. Thus, an individual who consumed a particular food 1 (1/7 = grade 0.14) to 2 (2/7 = grade 0.28) times per week had a final average score for that food of 0.21. This resulted in the following scores: 0 = <once/week; 0.21 = 1 to 2 times/week; 0.64 = 3 to 6 times/week; 1 = every day.

To continue with the analysis of this food database, it was necessary to reduce the list of 127 foods following criteria described in the literature (29–31). Foods were classified according to food groups as recommended by the food guide for the Brazilian population: fresh or minimally processed foods; processed; ultra-processed; oils, vitamins, salt, and sugar. Additionally, those foods for which low consumption was reported (consumption frequency less than 5%) were excluded from further analyses, making it possible to analyze a food consumption database consisting of 46 foods (29, 30).

### 2.2 Socioeconomic, psychosocial, and lifestyle questionnaire

The SpLS questionnaire is a generic instrument that was employed based on the literature (32–34) to collect information about the characteristics of the study population. The questionnaire was administered by a trained interviewer. This general questionnaire covered self-reported socioeconomic, lifestyle, and health issues, such as physical activity, alcoholism, smoking, anxiety, and stress.

In addition to the collection of this information, the Brazilian Food Insecurity Scale (BFIS) was administered. This is an instrument designed to assess, through 14 questions, concern about food shortages at home, including whether a respondent has gone without eating for an entire day within the last 3 months; is it applicable to families with or without household members under 18 years of age (32–34). After summing the scores obtained in relation to the InSan, these scores were categorized for this study as indicating food and nutritional security (SAN) or food and nutritional insecurity (InSAN) in order to contribute to the interpretation of the results.

Finally, to collect information on depressive symptoms, the Beck Depression Inventory II (BDI-II) was used (32). This questionnaire was answered individually by the participants, accompanied by a psychology professional to minimize bias and ensure peace of mind and safety for the participant. The BDI-II comprises a self-report questionnaire consisting of 21 items, with responses given on a scale from 0 to 3 points, to assess the presence and severity of depressive symptoms in normal and psychiatric populations. Higher scores indicate more severe depressive symptoms (35). In the present study, scores were grouped according to recommendations in the literature (35–38) into two categories: healthy or mild mood disorder (BDI-II <18), and depression (BDI-II ≥ 18).

### 2.3 Anthropometric measurements

Trained volunteers following standardized procedures measured participants’ height, weight, and waist circumference, as previously described by Lohman et al. (39) and the WHO (40).

Weight was measured using a Tanita® bioimpedance bipolar scale with 100 g increments and a capacity of 150 kg. The TBW® inextensible anthropometric tape (with 1.5 m length and 0.1 cm precision) was used to measure waist circumference. For men, waist circumference values above ≥102 cm were considered to represent a substantially increased risk of metabolic complications; for women, values ≥80 cm were considered to represent an increased risk and values ≥88 cm a substantially increased risk (40).

BMI (measured in kg/m^2^) was calculated using height and weight values for adults (20–59 years) (40). The definition of non-overweight BMI was a value below 24.9; participants with a BMI in the range from 25 to 29.9 were classified as overweight; and those with a BMI over 30 were classified as obese. Participants’ conicity index and waist–height ratio (WHtR) were calculated in order to investigate cardiovascular risk in the study population, using the mathematical equation proposed by Valdez et al. (41) for the former: conicity index = waist circumference (m)/0.109 × √body weight (kg)/height (m). The cutoff points suggested by Pitanga et al. (42) was used for classification of cardiovascular risk: ≥1.18 for women, and ≥ 1.25 for men. For WHtR, the reference value used was ≥0.5, with values above this threshold indicating higher cardiovascular risk (43).

### 2.4 Blood pressure measurement

For the measurement of blood pressure, the recommendations of the VI Brazilian Guidelines on Hypertension (44) were used. The instrument used for measurement was a G-TECH® premium blood aneroid sphygmomanometer. Blood pressure measurements were classified following the same guidelines (44).

### 2.5 Collection and analysis of blood samples

Blood collection was performed on the morning of the study after an overnight fast lasting between 7 and 8 h. All 10 mL samples were collected via venipuncture in disposable syringes. Blood samples were collected and individually divided into aliquots of 3 mL (collected in tubes with the anticoagulant ethylenediaminetetraacetic acid [EDTA]) for molecular analysis and 2 mL (collected in tubes containing the anticoagulant sodium fluoride [NaF]) for analysis of glucose. The remainder of each blood sample was transferred to an anticoagulant-free tube containing a separating gel for assessment of cortisol levels. The turbos were homogenized by inverting them 5 to 8 times and transported in refrigerated thermal boxes at a temperature of 2–8°C. Samples for biochemical analysis were kept at room temperature until clot retraction. Subsequently, they were centrifuged at 2500 rpm for 15 min.

Cortisol analysis was performed using the chemiluminescence method. Lower and upper thresholds of 6.7 μg/dL and 22.6 μg/dL, respectively, were considered as reference values for normal cortisol; cortisol values <6.7 μg/dL and > 22.6 μg/dL were considered altered (45).

Blood glucose was analyzed via the enzymatic colorimetric method (GOD-PAP), using a Glucose Monoregent K082 Bioclin^®^ kit according to the manufacturer’s instructions. The classification was based on the Guidelines of the Brazilian Society of Diabetes, 2014–2015 (46): values <65 mg/dL were classified as low, between 65 and 99 as normal, and > 100 mg/dL as high blood glucose.

Triglycerides, low-density lipoprotein (LDL), and high-density lipoprotein (HDL) were analyzed via enzymatic colorimetry using COD-PAP and specific colorimetric kits (Bioclin^®^, Belo Horizonte, Brazil) in a biochemical analyzer (Bioclin^®^ BS-120): K117 Bioclin^®^ Monoreagent Triglycerides, K083 Bioclin® Monoreagent Cholesterol, and K015 Bioclin^®^ Direct HDL. Low-density lipoprotein cholesterol (LDL-c) was quantified using the Friedewald equation (47). Reference values for lipid profiles were based on specifications by the Brazilian Society of Cardiology (SBC, 2017). Specifically, these were: LDL cholesterol, <160 mg/dL desirable, >160 mg/dL altered; HDL, <40 mg/dL altered, >40 mg/dL desirable; triglycerides, <150 desirable, >190 mg/dL altered.

To calculate the TyG index, the values for triglycerides (TG) and fasting glucose (GF) were considered using the following formula: Ln [Tg (mg/dL) × FG (mg/dL)/2], in which Ln represents the Neperian logarithm (48). This index was considered for the diagnosis of insulin resistance (IR), with threshold TyG index values of 4.68 for men and 4.55 for women (49).

### 2.6 Sample preparation, DNA extraction, and pyrosequencing reaction (PCR)

Three milliliters of each blood sample was transported to the Biotechnology Laboratory of the Exact, Natural, and Health Sciences Center of the Federal University of Espírito Santo (CCENS/UFES) and stored at-80°C until analysis. DNA extraction was performed using the leukocyte salt precipitation method (50). The quality of the extracted DNA was verified in a NanoDrop 2000/2000c spectrophotometer, and this verification was followed by methylation analysis. According to the manufacturer’s protocol, only 1 μg of DNA from each participant was converted to sodium bisulfite using the EZ DNA MethylationTM Kit (Zymo Research). The pyrosequencing technique was used for DNA methylation analysis (51, 52). The pyrosequencing methodology was followed after checking the quality of the PCR product on 2% agarose gels using GelRed (Uniscience). For this, PSQ 96 ID Pyrosequencer equipment (Qiagen, CA, United States) was used with the PyroMark Gold Q96 Reagent Kit (Qiagen), according to the manufacturer’s protocol. The *NR3C1* primer design and all pyrosequencing conditions were adapted from those used in previous research (51–54).

The *NR3C1* gene is located on chromosome 5 (q31–q32). The gene has seventeen exons, with eight coding exons (numbered from 2 to 9) and nine non-coding exons (1A to 1F and 1H to 1 J) that are located in the promoter region of the gene (21, 55). The promoter region is rich in CpG sequences, and the 1F region contains 47 CpG sites (53).

CpGs sites analyzed were amplified using forward primer (5′-TTTTTTTTTTGAAGTTTTTTTA-3′) and reverse primer (5′-BIOTIN-CCCCCAACTCCCCAAAAA-3′), which covered the region with 410 bp. Sequencing data were submitted to the GenBank database with accession number AY436590.1 (21).

The percentage of methylation was recorded for each CpG from 40 to 47, using the sequencing primers to CpG 40 to 42 (5′-AGAAAAGAAATTGGAGAAATT-3′) and CpG 43 to 47 (5′-GTTTTAGAGAGATTAGGT-3′). Two sequences were analyzed: Sequence 1 (YGGTGGTTTTTTTAAYGTYGTTTTAATCGTGTTGATCAGTCGCTTA) and Sequence 2 (YGGTTTTYGTYGTTGTYGTYGTTAGTCAGTTCAGTCGTAGTCAGTCGTA); these were analyzed using the PyroMark Q96 ID software 2.5, version 2.5.10.7.

### 2.7 Data analysis

The collected data were tabulated and submitted to consistency analysis. The Kolmogorov–Smirnov normality test was conducted in order to test the normality of the data. Tabulated data are presented in the form of the median (interquartile range); pairwise group comparisons were carried out using the Mann–Whitney U test. Continuous variables are presented in the form of the median with the interquartile range (IR); categorical variables are presented in the form of frequencies and percentages (%).

Pearson’s Chi-squared test and Fisher’s exact test were used to assess the associations between independent variables and the outcome of dietary intake patterns.

Dietary patterns were identified using principal component analysis (PCA), which was performed on z-score-transformed data (data were not normally distributed). The method was evaluated using the Kaiser–Meyer–Olkin (KMO) and Bartlett’s sphericity (BTS) tests. The evaluation criteria were a Kaiser–Meyer–Olkin (KMO) value greater than 0.6 and a value of p less than 0.05 for Bartlett’s test of sphericity. Eigenvalues >1.0 and the results of a scree test were used as criteria to determine how many distinct patterns to retain. Factor loadings >0.25 were considered statistically significant. Dietary patterns were named according to the food groups loading on each factor. It should be noted that this method requires complete data for all variables in the equation (29, 56, 57).

Variables representing individuals’ adherence to each dietary pattern were classified based on the factor scores generated after PCA and categorized into quartiles; these were further transformed into a dichotomous variable (yes or no), where variables in the 1st quartile corresponded to “does not consume” and variables in the 2nd, 3rd, and 4th quartiles corresponded to “does consume.”

Total methylation of the segment of interest (CpG 40 to 47) and the median percentage of methylation for each specific CpG site were analyzed. A factor analysis was conducted to explore the interrelationships between the CpGs within the segment using data from eight specific CpG sites. The main components extraction method was utilized due to the non-normality of the variables. Furthermore, multiple exploratory PCA was performed to investigate the relationship between the percentage methylation of the *NR3C1* gene and the separation of methylated CpG sites. The evaluation criteria included a KMO value >0.6 and a value of *p* < 0.05 for Bartlett’s test of sphericity. Eigenvalues >1.0 and the results of a scree test were used as criteria to determine how many components to retain. Factor loadings >0.25 were considered statistically significant. The factor loading matrix was estimated, and orthogonal rotation using the varimax method was conducted. This analysis resulted in two components: component 1 (CpG 44 to CpG 47) and component 2 (CpG 40 to CpG 43). Three different models were therefore considered overall: Model 1 (mean methylation of CpGs 40 to 47), Component 1 (average methylation of CpGs 44–47), and Component 2 (average methylation of CpGs 40–43).

The models were adjusted for various socioeconomic factors (gender, age, education, location, income), psychosocial factors (stress, anxiety, BFIS, BDI-II 18), nutritional status (TyG Index, HDL-c, LDL-c, BMI, cortisol), and lifestyle factors (physical activity, smoking, drinking). These confounding variables were selected based on examples from the literature and previous research conducted by the research group (29, 30, 38, 58, 59). A generalized linear model (GzLM) was utilized to analyze the data.

The GzLM offers advantages over the GLM in that it enables analysis of generalized linear models without data transformation, making it particularly suitable for non-parametric dependent variables (60, 61). The Akaike information criterion (AIC) was used to compare the models and identify the best-fitting model for the sample. The model incorporating the aforementioned confounding factors demonstrated the strongest fit to the data, as evidenced by the AIC.

The Statistical Package for the Social Sciences (SPSS), version 22 (IBM, Armonk, NY, United States), was used to analyze the data. GraphPad Prism^®^, version 7.0 (GraphPad® Software Inc., CA, United States), was used to generate graphical representations of the results. In all analyses, *p* < 0.05 were considered statistically significant.

## 3 Results

### 3.1 Dietary patterns

Three dietary patterns, as shown in Table 1, were derived. Their composition was as follows: “Pattern 1 – healthy”: beets, milk products, roots/stems/tubers, bananas, carrots, broccoli/cauliflower, tomatoes, leafy greens, cottage cheese/milk, cream, citrus fruits, papaya, okra, scarlet eggplant, apples, cucumber, boiled or sautéed onions, fruit candy, milkshake with fruit, homemade popcorn, beef or beef and pepper; “Pattern 2 – industrialized”: chocolate-based products, hot mixed sandwiches, sausages, wheat-derived products, sugary drinks, pumpkin, chayote; “Pattern 3 – mixed”: skimmed milk, pasta, wholegrain bread, cooked rice, farofa (a preparation with cornmeal and cassava flour).

**Table 1:** Food consumption patterns identified in the adult population living in the municipality of Alegre, Espirito Santo, Brazil.

Food	Dietary pattern		
	Healthy	Industrialized	Mixed
Beet	**0.597**		
Milk products	**0.583**	0.298	−0.290
Root/stem vegetables/tubers	**0.562**		
Bananas	**0.549**		
Carrots	**0.546**		
Broccoli/cauliflower	**0.509**		
Tomatoes	**0.500**		
Leafy greens	**0.498**	−0.334	
Cottage cheese/milk cream	**0.474**		−0.454
Citrus fruits	**0.454**		
Papaya	**0.418**	−0.301	−0.353
Okra	**0.410**	−0.381	
Scarlet eggplant	**0.382**	−0.274	
Apple	**0.368**		−0.300
Chocolate-based products	0.430	**0.479**	
Hot mixed sandwiches	0.286	**0.475**	−0.279
Sausages	0.394	**0.461**	
Wheat-derived products		**0.434**	
Sugarg drinks		**0.426**	0.302
Pumpkin	0.365	***−0.390***	0.268
Chayote	0.320	**−0.346**	
Skimmed milk			**−0.488**
Pasta			**0.443**
Wholegrain bread	0.259		**−0.423**
Cooked rice			**0.385**
Farofa (preparation with cornmeal)			**0.330**
Cucumber	**0.379**		
Homemade cake			
Boiled or sautéed onion	**0.310**		
Sauces	0.258		
Fruit candy	**0.302**		
Chocolate milk		**0.374**	
Whole milk			
Pork			
Milkshake with fruit	**0.372**		
Eggs	0.277		
Homemade popcorn	**0.311**		0.298
Cookies			
Beef or meat-based product	0.253	0.266	
Mango fruit or juice	0.258		
Pepper	**0.353**		
Chicken			
Tropeiro beans	**0.334**		
Baked beans			
Candies		0.290	
Cassava flour			**0.332**

PPSUS Project, 2016–2017. ^*^Foods with factor loadings ≥ 0.25 or ≤ − 0.25; total variance explained: 23.3%. The factor loadings of each component are shown in bold.

In the analysis, the “skimmed milk” component exhibited a high negative factor loading on the “mixed” pattern, indicating that individuals following this dietary pattern engaged in minimal consumption of skimmed milk.

The total variance explained by these factors was 23.3%. Foods with low loadings on any factor were considered to have weak correlations with overall dietary pattern and did not contribute significantly to any pattern. These included sauces, eggs, beef or meat-based product, mango fruit or juice, and candy/sweets; the findings indicated that these items were consumed consistently across individuals and did not align with any specific dietary pattern.

### 3.2 Effects of dietary pattern factors on participant outcomes

Two hundred and fifty participants were included in the cross-sectional analyses, comprising 21.2% (*n* = 53) men and 78.8% (*n* = 197) women. The median age within the sample was approximately 41 (IR = 32–50.25) years, and approximately 79.1% (*n* = 197) of the representative sample reported that they fell within the age range of 31–59 years.

Notably, 83% of these participants adhered to the “healthy pattern,” and there was a statistically significant association between adherence to this pattern and age (*p* = 0.008). Percentiles for each of the main continuous variables in relation to adherence to dietary patterns are shown in Table 2.

**Table 2:** Descriptive statistics on socioeconomic variables and nutritional status.

Variable		Adherence to healthy pattern		Value of *p*	Adherence to industrialized pattern		Value of *p*	Adherence to mixed pattern		*p*-value
	Total	No	Yes		No	Yes		No	Yes	
**Socioeconomic variables**										
*Gender, n (%)*										
Male	53 (21.2)	14 (22.6)	39 (20.7)	0.759	10 (16.1)	43 (22.9)	0.260	17 (27.4)	36 (19.1)	0.167
Female	197 (78.8)	48 (77.4)	149 (79.3)		52 (83.9)	145 (77.1)		45 (72.6)	152 (80.9)	
Age, median (IR)	41 (32; 50.25)	38 (28;49)	42 (34; 51)	0.098	43 (36; 52)	40 (32; 50)	0.133	44 (32; 52)	40 (33; 50)	0.368
*Age, n (%)*										
20–30 years	52 (20.9)	20 (32.8)	32 (17.0)	**0.008**	12 (19.7)	40 (21.3)	0.789	11 (17.70)	41 (21.9)	0.483
31–59 years	197 (79.1)	41 (67.2)	156 (83.0)		49 (80.3)	148 (78.7)		51 (82.3)	146 (78.1)	
*Marital status, n (%)*										
Single	58 (22.8)	18 (29.5)	40 (21.9)	0.244	13 (21.3)	45 (24.6)	0.602	12 (20.3)	46 (24.1)	0.477
Not single	186 (76.2)	43 (70.5)	143 (78.1)		48 (78.7)	138 (75.4)		47 (79.7)	139 (75.1)	
*Children, n (%)*										
No	56 (22.4)	17 (27.4)	39 (20.7)	0.274	15 (24.2)	41 (21.8)	0.696	12 (19.4)	44 (23.4)	0.507
Yes	194 (77.6)	45 (72.6)	149 (79.3)		47 (75.8)	147 (78.2)		50 (80.6)	144 (76.6)	
Years of education, median (IR)	8 (4; 11)	8 (4; 11)	8 (4; 11)	0.612	4 (4; 8)	8 (4; 11)	**0.020**	8 (4; 11)	8 (4; 11)	0.188
*Education, n (%)*										
Below 8 years	97 (39.6)	22 (36.1)	75 (40.8)	0.516	35 (57.4)	62 (33.7)	**0.001**	22 (36.7)	75 (40.5)	0.594
More than 8 years	148 (60.4)	39 (63.9)	109 (59.2)		26 (42.6)	122 (66.3)		38 (63.3)	110 (59.5)	
Income classification, median (IR)	5.00 (3.34; 8.55)	4.27 (3.21; 8.97)	5.04 (3.34; 8.55)	0.339	3.63 (2.40; 8.55)	5.70 (3.42; 8.94)	**0.010**	5.04 (3.34; 8.55)	5.01 (3.34; 8.90)	0.870
*Income classification, % (n)*										
<$5.00/day	132 (53.0)	34 (55.7)	98 (52.1)	0.624	39 (63.9)	93 (49.5)	**0.049**	34 (54.8)	98 (52.4)	0.739
>$5.00/day	117 (47.0)	27 (44.3)	90 (47.9)		22 (36.1)	95(50.5)		28 (45.2)	89 (47.6)	
*Location of residence, n (%)*										
Rural	88 (35.3)	29 (46.8)	59 (31.6)	**0.030**	28 (45.2)	60 (32.1)	0.062	26 (42.6)	62 (33.0)	0.171
Urban	161 (64.7)	33 (53.2)	128 (68.4)		34 (54.8)	127 (67.9)		35 (57.4)	126 (67.0)	
*Nutritional status*										
Cortisol, median (IR)	11.35 (8.47; 14.32)	12.6 (9.5; 14.4)	11.20 (8.10;14.30)	0.195	12.60 (8.70; 14.10)	11.20 (8.20;14.40)	0.401	11.00 (8.50; 13.70)	11.40 (8.40; 14.50)	0.635
*Cortisol, % (n)*										
Normal	215 (89.2)	54 (90.0)	161 (89.0)	0.820	53 (91.4)	162 (88.5)	0.541	55 (93.2)	160 (87.9)	0.253
Altered	26 (10.8)	6 (10.0)	20 (11.0)		5 (8.6)	21 (11.5)		4 (6.8)	22 (12.1)	
Glucose, median (IR)	92 (84. 4)	89 (82; 100)	91 (82; 104)	**0.039**	91 (82; 97)	93.5 (86; 105)	**0.046**	94 (88; 103)	92 (83; 103)	0.327
*Glucose, n (/%)*										
Normal	168 (68.3)	45 (72.6)	123 (66.8)	0.401	48 (77.4)	120 (65.2)	0.074	41 (67.2)	127 (68.6)	0.834
Altered	78 (31.7)	17 (27.4)	61 (33.2)		14 (22.6)	64 (34.8)		20 (32.8)	58 (31.4)	
Triglycerides, median (IR)	121 (87.0; 175.0)	108.50 (79.00; 162.00)	124.00 (93.00;175.00)	0.100	117.00 (83.00; 174.00)	122.00 (90.00;175.00)	0.692	116.00 (85.00;146.00)	128.00 (87.00;180.00)	0.260
*Triglycerides, n (%)*										
Normal	163 (66.0)	44 (71.0)	119 (64.3)	0.339	41 (67.2)	122 (65.6)	0.617	46 (75.4)	117 (62.9)	0.074
Altered	84 (34.0)	18 (29.0)	66 (35.7)		20 (32.8)	64 (34.4)		15 (24.6)	69 (37.1)	
TyG index, median (IR)	4.68 (4.49; 4.90)	4.59 (4.40;4.82)	4.69 (4.52;4.91)	**0.042**	4.62 (4.45; 4.85)	4.69 (4.49; 4.91)	0.427	4.65 (4.50; 4.76)	4.69 (4.48; 4.92)	0.451
*Insulin resistance, n (%)*										
No	86 (35.1)	26 (41.9)	60 (32.8)	0.192	23 (37.7)	63 (34.2)	0.623	23 (37.7)	63 (34.2)	0.623
Yes	159 (64.9)	36 (58.1)	123 (67.2)		38 (62.3)	121 (65.8)		38 (62.3)	121 (65.8)	
HDL-c, median (IR)	66.0 (53.0; 78.0)	67.00 (47.00; 78.00)	66.00 (55.00; 78.00)	0.484	64.00 (48.00; 77.00)	67.43 (54.00; 79.00)	0.146	68.00 (58.00; 80.00)	65.00 (52.00; 78.00)	0.144
*HDL-c, n (%)*										
Normal	220 (89.1)	53 (85.5)	167 (90.3)	0.296	51 (83.6)	169 (90.9)	0.115	57 (93.4)	163 (87.6)	0.207
Altered	27 (10.9)	9 (14.5)	18 (9.7)		10 (16.4)	17 (9.10)		4 (6.6)	23 (12.4)	
LDL-c, median (IR)	85.0 (69.0; 110.0)	84.50 (68.19; 111.00)	87.00 (70.00; 110.00)	0.636	84.00 (66.00; 108.00)	86.50 (70.00; 110.00)	0.691	83.00 (66.00; 110.20)	86.50 (71.00; 109.00)	0.518
^**^LDL-c, n (%)										
Normal	244 (98.8)	62 (100.0)	182 (98.4)	0.575	60 (98.4)	184 (98.9)	0.575	60 (98.4)	184 (98.9)	0.575
Altered	3 (1.2)	0 (0.0)	3 (1.6)		1 (1.6)	2 (1.1)		1 (1.6)	2 (1.1)	
BMI, median (IR)	27.5 (24.1)	26.35 (24.28; 30.11)	27.81 (23.88; 32.34)	0.298	27.43 (24.02; 30.45)	27.43 (24.02; 30.45)	0.684	25.76 (23.25; 32.14)	27.65 (24.37; 31.56)	0.457
*BMI, n (%)										
Underweight/eutrophy	80 (32.7)	21 (34.4)	59 (32.1)	0.172	19 (31.7)	61 (33.0)	0.625	23 (37.7)	57 (31.0)	0.364
Overweight	82 (33.5)	25 (41.0)	57 (31.0)		23 (38.3)	59 (31.9)		16 (26.2)	66 (35.9)	
Obese	83 (33.9)	15 (24.6)	68 (37.0)		18 (30.0)	65 (35.1)		22 (36.1)	61 (33.2)	
Waist circumference, median (IR)	91 (81; 101)	88.00 (81.00; 98.00)	93.00 (81.00; 102.00)	0.177	91.00 (80.00; 100.00)	92.00 (81.00; 101.00)	0.792	91.50 (79.00; 100.00)	91.50 (79.00; 100.00)	0.636
*Waist circumference, n (%)*										
Low risk	72 (29.1)	21 (34.4)	51 (27.4)	0.296	18 (29.0)	54 (29.2)	0.981	20 (33.3)	52 (27.8)	0.412
High risk	175 (70.9)	40 (65.6)	135 (72.6)		44 (71.0)	131 (70.8)		40 (66.7)	135 (72.2)	
Conicity index, median (IR)	1.25 (1.19; 1.31)	1.25 (1.16; 1.31)	1.25 (1.20; 1.31)	0.458	1.25 (1.19; 1.33)	1.25 (1.20; 1.30)	0.415	1.25 (1.19; 1.30)	1.25 (1.20; 1.31)	0.627
*Conicity index classification, n (%)*										
Risk indicator	176 (71.3)	39 (63.9)	137 (73.7)	0.145	44 (71.0)	132 (71.4)	0.954	42 (70.0)	134 (71.7)	0.805
No risk indicator	71 (28.7)	22 (36.1)	49 (26.3)		18 (29.0)	53 (28.6)		18 (30.0)	53 (23.3)	
Waist–height ratio, median (IR)	0.56 (0.5; 0.63)	0.54 (0.50; 0.62)	0.57 (0.50; 0.63)	0.228	0.57 (0.50;0.66)	0.57 (0.50; 0.66)	0.499	0.56 (0.49; 0.62)	0.56 (0.51; 0.63)	0.429
*Waist–height ratio classification, n (%)*										
High cardiovascular risk	66 (26.7)	19 (31.1)	47 (25.3)	0.368	17 (27.4)	49 (26.5)	0.886	19 (31.7)	47 (25.1)	0.320
Low cardiovascular risk	181 (73.3)	42 (17.0)	139 (74.7)		45 (72.6)	136 (73.5)		41 (68.3)	140 (74.9)	
*Blood pressure, n (%)*										
Normal	195 (81.9)	51 (86.4)	144 (80.4)	0.299	47 (79.7)	148 (82.7)	0.601	50 (86.2)	145 (80.6)	0.331
Altered	43 (18.1)	8 (13.6)	35 (19.6)		12 (20.3)	31 (17.3)		8 (13.8)	35 (19.4)	
**Psychosocial variables**										
*Stress, n (%)*										
No	96 (38.4)	27 (43.5)	69 (36.7)	0.336	19 (30.6)	77 (41.0)	0.148	21 (33.9)	75 (39.9)	0.398
Yes	154 (61.6)	35 (56.5)	119 (63.3)		43 (69.4)	111 (59.0)		41 (66.1)	113 (60.1)	
*Anxiety, n (%)*										
No	84 (33.6)	23(37.1)	61 (32.4)	0. 501	16 (25.8)	68 (36.2)	0.134	22 (35.5)	62 (33.0)	0.717
Yes	166 (66.4)	39 (62.9)	127 (67.6)		46 (74.2)	120 (63.8)		40 (64.5)	126 (67.0)	
BFIS	0 (0;4)	0 (0;4)	0 (0; 3)	0.670	1 (0; 4)	0 (0; 3)	0.279	0 (0; 4)	0 (0; 3)	0.886
*BFIS rating, n (%)*										
Food safety	148 (60.2)	40 (64.5)	108 (58.7)	0.418	35 (57.4)	113 (61.1)	0.608	38 (62.3)	110 (59.5)	0.695
Food insecurity	98 (39.8)	22 (35.5)	76 (41.3)		26 (42.6)	72 (38.9)		23 (37.7)	75 (40.5)	
Beck total score	8 (4; 15)	7 (4; 12)	8 (4; 16)	0.469	9.5 (3.0;17.0)	8.0 (4.0; 14.0)	0.479	7.0 (3.0; 14.0)	9.0 (4.0; 15.0)	0.417
*BDI II-18, n (%)*										
No depressive symptoms	181 (78.7)	46 (86.8)	135 (76.3)	0.101	44 (75.9)	137 (79.0)	0.542	47 (79.7)	134 (78.4)	0.834
Depressive symptoms	49 (21.3)	7 (13.2)	42 (23.7)		14 (24.1)	35 (20.3)		12 (20.3)	37 (21.6)	
*Smoker, n (%)*										
No	230 (92.4)	58 (93.5)	172 (92.0)	0.790	57 (91.9)	173 (92.5)	0.882	57 (91.9)	173 (92.5)	0.882
Yes	19 (7.6)	4 (6.5)	15 (8.0)		5 (8.1)	14 (7.5)		5 (8.1)	14 (7.5)	
*Alcohol consumption, n (%)*										
No	165 (66.5)	41 (66.1)	124 (66.7)	0.938	42 (67.7)	123 (66.1)	0.816	39 (63.9)	126 (67.4)	0.620
Yes	83 (33.5)	21 (33.9)	62 (33.3)		20 (32.3)	63 (33.9)		22 (36.1)	61 (32.6)	
*Engage in physical activity, n (%)*										
No	159 (64.6)	36 (61.0)	123 (65.8)	0.505	42 (71.2)	117 (62.6)	0.227	46 (75.4)	113 (61.1)	**0.042**
Yes	87 (35.4)	23 (39.0)	64 (32.2)		17 (28.8)	70 (37.4)		15 (24.6)	72 (38.9)	

TyG index, triglyceride–glycemic index; HDL-c, high-density lipoprotein; LDL-c, low-density lipoprotein; BMI, body mass index; BDI-II, beck depression inventory. ^*^BMI, after Bonferroni correction, the new *p*-value was 0.008. Comparison using Kruskal–Wallis test showed a significant result (*p* < 0.05). For insulin resistance (RI) and glucose levels (Glic), Mann–Whitney tests indicated a significant difference (*p* < 0.05). ^**^Fisher’s exact test was used for a variable with cell count <5, yielding a significant result. In bold are significant *p*-values.

The location variable stood out among the socioeconomic variables evaluated in this study. The majority of participants (*n* = 161) lived in urban areas, and 68.4% of these (*n* = 128) adhered to the healthy dietary pattern. An association test revealed a significant relationship between location and dietary pattern (*p* = 0.030).

There was a significant association between the healthy pattern and the following biochemical parameters: glucose levels (*p* = 0.039) and TyG index (triglyceride-glucose) (*p* = 0.042). Individuals who adhered to the healthy eating pattern had significantly higher median values. This indicates a stronger central tendency among individuals following a healthy diet.

The sample population overall showed altered anthropometric measurements (BMI, waist circumference, conicity index) according to health guidelines, as shown in Table 2. However, there were no significant differences between the groups (*p* > 0.005) in terms of these measurements. Furthermore, most participants (*n* = 159) reported a lack of physical activity, indicating a sedentary lifestyle.

Food consumption following the “industrialized pattern” exhibited a significant relationship (*p* < 0.05) to socioeconomic variables: specifically, there were differences between groups classified according to years of education (*p* = 0.020) and income (*p* = 0.010). Most individuals with more than 8 years of education (*n* = 122) adhered to the “industrialized pattern,” with an association test indicating dependence between the variables (*p* = 0.001). Regarding income, most participants (*n* = 95) who had an income of less than 5 dollars per day adhered to the industrialized pattern (*p* = 0.049). Of the biochemical variables analyzed, glucose was the only one for which significantly different median values were observed in relation to this dietary pattern (*p* < 0.05).

In this case it would be 64.6%, in this question some patients did not respond, the total number of patients in this question is 246. Representing some losses in respondents.

### 3.3 Definition of the main component factors of *NR3C1* methylation

The interaction between the CpGs and the model generated by the extraction of two main components (component 1: CpG44-45-46-47; component 2: CpG40-41-42-43) was submitted to a factor analysis, with 72.2% of the total variation in the segment explained (Supplementary Figure S1). The total methylation was given by the mean of the methylation percentages for the CpG segment 40–47 and the methylation of components 1 and 2.

### 3.4 Associations between sequence methylation, methylation in component 1 and component 2 of the 1F region of *NR3C1*

Multivariate GzLM analysis was conducted to verify the relationship between the mean methylation, component 1 methylation (CpG44 to CpG47), and component 2 methylation (CpG40 to CpG43) of the *NR3C1* gene segment after inclusion of confounding variables relating to socioeconomic factors (gender, age, education, location, marital status, children, income), nutritional and biochemical status (HDL and LDL cholesterol, cortisol, BMI, TyG index), psychosocial stress (Beck Depression Inventory, Food and Nutrition Insecurity, stress, and anxiety), lifestyle (physical activity, smoking, and alcohol consumption), and food consumption pattern.

The results of the multivariate analysis are presented in Table 3, providing information about the relationship between these variables and the *NR3C1* gene segment.

**Table 3:** GzLM results for the association of methylation at the 1F region of NR3C1 (sequence mean and methylation at components 1 and 2) with independent variables relating to socioeconomic status, nutritional status, lifestyle, and food consumption.

	Sequence mean*					Component 1 methylation					Component 2 methylation				
Variable	B	Std. error	95% CI		Value of *p*	B	Std. error	95% CI		Value of *p*	B	Std. error	95% CI		Value of *p*
			Lower	Upper				Lower	Upper				Lower	Upper	
*Gender*															
Male	1.261	0.416	0.445	2.077	**0.002**	−1.253	0.139	−1.525	−0.980	**0.000**	−1.017	0.531	−2.057	0.023	0.055
Female															
Age	−0.005	0.011	−0.027	0.017	0.662	−0.076	0.007	−0.089	−0.063	**0.000**	−0.021	0.012	−0.044	0.002	0.075
*Marital status*															
Single	0.774	0.396	−0.003	1.551	0.051	3.009	0.216	2.586	3.431	**0.000**	0.833	0.404	0.042	1.625	**0.039**
Not single															
*Children*															
No	−0.174	0.449	−1.054	0.707	0.699	−6.175	0.269	−6.703	−5.648	**0.000**	0.477	0.396	−0.299	1.253	0.228
Yes															
Education (years)	−0.104	0.037	−0.177	−0.031	**0.005**	−0.236	0.020	−0.275	−0.197	**0.000**	−0.121	0.064	−0.246	0.005	0.061
*Per capita* income (USD/day)	−0.019	0.014	−0.046	0.008	0.171	−0.162	0.008	−0.177	−0.147	**0.000**	−0.011	0.012	−0.035	0.013	0.366
BMI	−0.101	0.018	−0.135	−0.066	**0.000**	−0.234	0.005	−0.245	−0.224	**0.000**	−0.012	0.019	−0.048	0.025	0.531
TyG index	0.079	0.344	−0.594	0.752	0.818	0.872	0.098	0.679	1.064	**0.000**	1.158	0.408	0.357	1.958	**0.005**
HDL_c (mg/dL)	0.007	0.005	−0.003	0.018	0.171	0.034	0.003	0.029	0.039	**0.000**	0.018	0.005	0.008	0.028	**0.000**
LDL_c (mg/dL)	0.005	0.005	−0.004	0.014	0.239	0.005	0.002	0.001	0.010	**0.014**	0.013	0.004	0.004	0.022	**0.004**
Cortisol (μg/dL)	0.014	0.023	−0.030	0.058	0.528	0.072	0.013	0.048	0.097	**0.000**	−0.025	0.025	−0.073	0.023	0.314
*Stress*															
No	0.224	0.244	−0.255	0.702	0.360	2.997	0.120	2.762	3.232	**0.000**	−0.019	0.182	−0.377	0.338	0.915
Yes															
*Anxiety*															
No	0.138	0.254	−0.359	0.636	0.585	−2.254	0.110	−2.470	−2.038	**0.000**	0.016	0.289	−0.550	0.581	0.957
Yes															
BFIS	0.033	0.028	−0.022	0.088	0.233	−0.004	0.014	−0.032	0.024	0.768	0.029	0.021	−0.012	0.071	0.166
Beck total score	−0.002	0.014	−0.029	0.025	0.878	−0.072	0.008	−0.088	−0.056	**0.000**	−0.018	0.010	−0.038	0.001	0.062
*Physical activity*															
No	−0.221	0.251	−0.712	0.270	0.378	−1.857	0.090	−2.032	−1.681	**0.000**	−0.370	0.278	−0.915	0.175	0.183
Yes															
*Alcohol consumption*															
No	0.610	0.318	−0.013	1.233	0.055	3.697	0.136	3.430	3.963	**0.000**	0.028	0.279	−0.519	0.575	0.919
Yes															
*Smoking*															
No	−0.378	0.761	−1.870	1.115	0.620	−7.058	0.367	−7.777	−6.338	**0.000**	−3.728	1.467	−6.602	−0.853	**0.011**
Yes															
*Adherence to healthy pattern*															
No	−0.839	0.362	−1.549	−0.129	**0.021**										
Yes															
*Adherence to industrialized pattern*															
No						−1.279	0.190	−1.651	−0.908	**0.000**					
Yes															
*Adherence to mixed pattern*															
No						−2.380	0.111	−2.598	−2.163	**0.000**	−0.471	0.303	−1.066	0.123	0.120
Yes															

TyG index, triglyceride-glycemic index; HDL-c, high-density lipoprotein; LDL-c, low-density lipoprotein; BMI, body mass index; BDI-II, beck depression inventory. *The models for the GzLM analysis were adjusted and selected based on the Akaike Information Criterion (AIC). Three separate models were defined for the sequence mean (CpG40-47), component 1 (CpG44-45-46-47), and component 2 (CpG40-41-42-43) outcomes. The AIC was used to determine the model that best fit the data, considering both complexity and goodness of fit. O *p*-value significant was apresentado em negrito (*p* < 0.05).

The results revealed significant associations between mean sequence methylation and variables including sex, years of education, BMI, and adherence to the healthy dietary pattern (*p* < 0.05). These relationships were observed in the final model, which was determined based on comparison of Akaike information criterion (AIC) values. The model with the lowest AIC value was considered to represent the best fit (with the most suitable adjustments) for the sample.

Table 3 presents the output of the final model, highlighting the variables that showed statistically significant associations with mean sequence methylation and the strength of these associations. This model, which included the confounding factors mentioned previously, showed the greatest fit to the data.

Compared to men, overall methylation in the *NR3C1* gene segment (CpG 40 to CpG 47) was increased by 1.261 times in women (*β* = 1.261, *p* = 0.002, 95% Wald CI [0.445; 2.077]). Each year of education was associated with a 0.104-fold reduction in methylation (*β* = −0.104, *p* = 0.005, 95% Wald CI [−0.177; −0.031]). For each unit increase in BMI, methylation was decreased by 0.101 (*β* = −0.101, *p* = 0.000, 95% Wald CI [−0.135, −0.066]); and not adhering to the “healthy” dietary pattern was associated with a 0.839-fold reduction in methylation (*β* = −0.839, *p* = 0.021, 95% Wald CI [−0.149, −0.129]).

The results (Table 3) also indicated that relationships were present between methylation in component 1 of the segment (CpG 44 to CpG 47) and the variables of sex, age, marital status, children, education, *per capita* income, BMI, TyG index, HDL and LDL cholesterol, cortisol, stress, anxiety, total Beck score, physical activity, alcohol consumption, smoking, and adherence to the industrialized and mixed dietary patterns, with each of these associations showing statistical significance in the final model (*p* < 0.05). Specifically, being male was associated with a 1.253-fold reduction (*β* = −1.261, *p* = 0.000, 95% Wald CI [−1.525; −0.980]) in methylation in component 1 of the *NR3C1* gene (CpG 44 to CpG 47). Each year of age was associated with a 0.076-fold reduction in methylation (*β* = −0.076, *p* = 0.000, 95% Wald CI [−0.089; −0.063]). Not being married was associated with a 3.009-fold increase in methylation (*β* = 3.009, *p* = 0.000, 95% Wald CI [2.586; 3.431]), and not having children was associated with a 6.175-fold increase (*β* = −6.175, *p* = 0.000, 95% Wald CI [−6.703; −5.648]). Regarding education, each year of education was associated with a reduction in methylation by 0.236 times (*β* = −0.236, 95% Wald CI [−0.275;-0.197]). Each unit increase in *per capita* income was associated with a reduction in methylation in component 1 by 0.162 (*β* = −0.162; *p* = 0.000, 95% Wald CI [−0.177;-0.147]). Each unit increase in BMI was associated with a decrease in methylation by 0.234 times (*β* = −0.234; *p* = 0.000, 95% Wald CI [−0.245–0.224]). Regarding the biochemical parameters, each unit increase in TyG index was associated with an increase in methylation by 0.872 times (*β* = 0.872; *p* = 0.000, 95% Wald CI [0.679; 1.064]). However, each unit increase in HDL cholesterol was associated with an increase in methylation by 0.034 times (*β* = 0.034; *p* = 0.000, 95% Wald CI [0.029; 0.039]), and each unit increase in LDL was associated with a 0.005-fold increase in component 1 methylation (*β* = 0.005; *p* = 0.014, CI 95% Wald [0.001; 0.010]). Increased cortisol levels were associated with a 0.072-fold increase in methylation (*β* = 0.072; *p* = 0.000, 95% Wald CI [0.048; 0.097]). Not being stressed was associated with a 2.997-fold increase in methylation (*β* = 2.997; *p* = 0.000, 95% Wald CI [2.762; 3.232]), and not being anxious was associated with a 2.254-fold reduction in methylation (*β* = 0.072; *p* = 0.000, 95% Wald CI [0.048; 0.097]). Each unit increase in total Beck Inventory score was associated with a 0.072-fold reduction in methylation (*β* = −0.072; *p* = 0.000, 95% Wald CI [−0.088; 0.056]). Not engaging in physical activity was associated with a reduction in methylation by 1.857 times (*β* = −1.857; *p* = 0.000, 95% Wald CI [−2.032; −1.681]). In relation to lifestyle, not consuming alcohol was associated with an increase in methylation by 3.697 times (B = 3,697, *p* = 0.000, 95% Wald CI [−2.032; 3.963]), while not smoking was associated with a reduction in methylation by 7.058 times (*β* = −7.058, p = 0.000, 95% Wald CI [−7.777, −6.338]). Finally, in the model with component 1 methylation as the outcome variable, non-adherence to the “industrialized” dietary pattern was found to be associated with a reduction in methylation by 1.279 times (*β* = −1.279, *p* = 0.000, 95% Wald CI [−1.651, −0.908]). Similarly, not adhering to the “mixed” pattern was associated with a 2.380-fold reduction in methylation (*β* = −2.380, *p* = 0.000, 95% Wald CI [−2.598, −2.163]).

The results additionally indicated that relationships were present between methylation in component 2 (CpG 40 to CpG 43) and the variables of marital status, Tyg index, HDL and LDL cholesterol, and smoking (*p* < 0.05), with these associations showing statistical significance in the final model and meeting the GzLM criteria (Table 2). Regarding marital status, not being married was associated with an increase in methylation in component 2 of the *NR3C1* gene (CpG 40 to CpG 47) by 0.833 times (*β* = 0.833, *p* = 0.039, 95% Wald CI [0.042; 1.625]). Each unit increase in TyG index was associated with a 1.158-fold increase in methylation (*β* = 1.158, *p* = 0.005, 95% Wald CI [0.357; 1.958]). Regarding lipid profile, each unit increase in HDL was associated with an increase in methylation by 0.018 (*β* = 0.018, *p* = 0.000, 95% Wald CI [0.008; 0.028]), and each unit increase in LDL with an increase in methylation by 0.013 times (*β* = 0.013, *p* = 0.004, 95% Wald CI [0.004; 0.022]). In terms of lifestyle, the smoking variable was the only one that achieved significance under this model: specifically, not smoking was associated with a decrease in methylation by 3,728 times (*β* = −3,728, *p* = 0.011, 95% Wald CI [−6,602; −0,853]).

## 4 Discussion

In the present study, three distinct dietary patterns were identified among a sample of 250 patients seen in primary health care settings; their classification was based on recommendations and the specialized literature (28–30). The analyses conducted to assess the correlation between *NR3C1* methylation and food consumption were prompted by the significant impact of “environmental stress” or “metabolic stress,” both widely recognized as contributors to DNA methylation (10–12).

Surprisingly, the scientific literature has yet to thoroughly examine the effects of distinct dietary patterns, either individually or in combination, on epigenetic modifications within the *NR3C1* gene. Existing studies in this domain have predominantly centered around isolated nutrients or investigated the interplay of other genes with the one-carbon cycle (26, 61).

In our study, the association between methylation in region 1F (CpG 40–47) and the consumption of industrialized patterns persisted as a significant finding even after meticulous adjustment for biochemical, anthropometric, psychosocial, and socioeconomic confounding factors. Additionally, we noted a potentially relevant relation with mean NR3C1 DNA methylation in the CpGs 40-43 and 44-47, revealing noteworthy implications for BMI, serum cortisol levels, and lipid profile.

Three distinct patterns of consumption were identified in our population (healthy, industrialized, and mixed). These patterns reflect the socioeconomic context of the region and differ from international consumption patterns (13–15, 62, 63).

Short-and long-term changes in dietary patterns are correlated with epigenetic modifications, and particularly with DNA methylation, thereby influencing the expression of pivotal genes crucial for maintaining metabolic balance. Nicoletti et al. (64) conducted a short-term hypocaloric intervention for women with severe obesity, and observed restoration of the obesity-related DNA methylation pattern proportional with the rate of weight loss following the dietary intervention. Similarly, Barchitta et al. (63) observed a group of women and found that high adherence to a Western (industrialized) dietary pattern was associated with lower LINE-1 methylation compared to that occurring in those following a “prudent” (healthy) dietary pattern. LINE-1 methylation serves as a marker for global genomic DNA methylation, which is linked to conditions such as cancer and cardiovascular, and neurodegenerative diseases. This outcome implies that low methylation levels are correlated with a heightened risk of developing chronic diseases.

The authors of one study have proposed that chronic stress has a detrimental impact on eating behaviors. Participants experiencing chronic stress were found to be more prone to engaging in unhealthy eating behaviors, and this association was mediated by depressive symptoms rather than current smoking status (65). Another study conducted in Chile found that, although chronic stress and diet quality did not act as mediators in the relationship between weight stigma and BMI, there was a significant association between weight stigma and heightened chronic stress, along with lower diet quality—factors previously found to be associated with BMI (66).

In this context, the complexity of the food process is underscored, particularly as “metabolic stress” emerges as a crucial factor contributing to molecular and epigenetic changes. These alterations are primarily linked to the consumption of large quantities of sugars and fats, especially in connection with the intake of ultra-processed foods (10–12).

In experimental studies conducted by Berry et al. (10), animals underwent exposure to two stressors—one during the gestational phase and another during adulthood, both associated with metabolic stress induced by a high-fat diet. The researchers observed a reduction in the expression of genes related to stress response and inflammation, namely *NR3C1* and *FKBP5*, in adult animals affected by prenatal stress and in a metabolic stress group subjected to a high-fat diet. The authors proposed a comparison between the impact of a diet rich in ultra-processed foods and prenatal stress.

Along similar lines, our study indicated that adherence to an industrialized diet increases methylation in the CpGs of component 1 (44–47) of the *NR3C1* gene, confirming the initial hypothesis. The *NR3C1* gene, which encodes GR, plays an important role in regulating activation of the HPA axis, modulating the physiological impact of environmental stress, and regulating glucocorticoid production (10).

Changes in *NR3C1* gene methylation in the segment analyzed have previously been found to be related to certain psychiatric disorders, such as depression (28) and post-traumatic stress (67, 68), eating disorders (69), abuse, and trauma or adverse events in childhood or during the prenatal period (53, 70, 71). In addition, methylation of the *NR3C1* gene has been reported to reduce GR transcript levels and GR protein levels, and to be associated with altered cortisol levels and stress reactivity (72).

Habits, behaviors, lifestyle, and health status can influence *NR3C1* gene regulation through methylation, expressing the complexity of environmental effects in the form of stress reactivity (73), as well as inducing chronic stress, which has also been found to be associated with *NR3C1* epigenetic changes (38). Prolonged stress can affect control of the HPA axis via negative feedback within the GR–cortisol complex and can cause changes in stress reactivity, such as hyperactivation of the axis or depletion with hypoactivation (16, 59, 70).

Being overweight has been found to be associated with both hyperactivation of the HPA axis and, paradoxically, hypocortisolemia (59). This paradoxical response suggests that prolonged exposure to chronic high stress may indicate diminished adaptability of the primary neurobiological stress response system, resulting in reduced cortisol responsiveness and hypocortisolemia (71). Our results revealed a correlation between elevated cortisol levels, adherence to the industrialized dietary pattern, and increased methylation of *NR3C1* component 1.

The *NR3C1* gene is also involved in regulation of the immune and metabolic systems, through the expression of GR and the action of GR as a transcription factor in glucocorticoid response elements and via non-genomic mechanisms (16). Thus, epigenetic changes in *NR3C1* associated with an industrialized diet can interfere with physiological, metabolic, and immunological modulation, activating the detrimental effects of increased intake (74). In this study, we observed biochemical changes, such as alterations in HDL-c and LDL-c, glucose, and TyG index, which exhibited a positive association with methylation in component 1 of the *NR3C1* gene and with adherence to the industrialized dietary pattern.

In our study, we incorporated food consumption into our models as a lifestyle factor in order to understand its role in environmental influence on *NR3C1* gene methylation. Building on findings from Assis Pinheiro et al. (73), our study integrated lifestyle variables and revealed associations of *NR3C1* gene methylation with alcohol consumption, overweight, and high cortisol levels. These factors were linked to non-methylation of *NR3C1*, while depression was associated with methylation, resulting in unique clinical implications. Changes in DNA methylation were found to be accompanied by alterations in anxiety-like behaviors and increased stress response (75). Reduced efficiency of glucocorticoid signaling contributes to increased inflammation, immune processes, and various health-related issues, including behavioral pathologies, insulin resistance, bone metabolism, and the acquired immune response (76).

Finally, all of the parameters associated with adherence to an industrialized (ultra-processed) diet resulted in epigenetic changes in *NR3C1*, a gene related to chronic stress, stress responsiveness, and depression. To this end, the present study used a combination of robust statistical models first to define food consumption patterns and subsequently input them into Generalized Linear Models with advantages of allowing the admitted assumptions and examine the linear relationships between the explanatory variables and the response.

We recognize the potential limitations in using the Food Frequency Questionaire (FFQ) instrument, common in population studies, which may pose challenges for the estimation of components of respondents’ diet. The instrument’s extensive list of food items could lead to confusion and memory bias among respondents, limiting its utility (28). However, to mitigate potential errors, the study followed recommendations in the literature (28–30), including providing training to those who administered the instrument. Additionally, a widely accepted instrument was utilized in this study in which food items were condensed into consumption patterns, aligning with the reality of the study population and facilitating comprehensive analysis.

The findings highlighted the correlation between chronic stress and flattened serum cortisol levels, suggesting a potential link to primary or chronic adrenocortical insufficiency due to hyperactivation or chronic stress. Methylation of the *NR3C1* gene in its promoter region is connected to diminished expression of the glucocorticoid receptor (GR) and to disruption of the hypothalamic–pituitary–adrenal (HPA) axis, and is implicated in the development of psychiatric disorders among individuals exposed to psychosocial stress, early trauma, post-traumatic stress, gestational hunger, neglect, and various other adverse conditions (55, 77, 78). Despite these well-established associations, particular emphasis has been placed on readily accessible peripheral tissues in studies examining life adversity, weight accumulation, associated comorbidities, and lifestyle (38, 58, 64, 73). The utilization of peripheral methylation as a substitute for methylation in hippocampal cells, including the analysis of samples from individuals who died by suicide, is grounded in the notion that methylation patterns at specific loci may exhibit consistency between the brain and the periphery, indicating epigenetic reprogramming associated with psychological conditions (55). The correlation between DNA methylation in genes related to the hypothalamic–pituitary–adrenal (HPA) axis and peripheral origin, along with indicators of neural function and perceived daily stress (79), remains stable for up to 2 years. Furthermore, a robust correlation exists with methylation in other tissues. Circulating leukocytes can effectively mirror DNA methylation patterns in adipose tissue related to obesity, offering valuable biomarkers for obesity diagnosis through minimally invasive peripheral blood analysis (80), and in neuronal cells located in the hippocampus (81).

In a study conducted in Brazil, an association was discovered between peripheral methylation of the NR3C1 gene and glycemic levels, as well as insulin resistance. Noteworthy factors were found to include age, smoking, body mass index (BMI), and methylation in the CpG40 and CpG43 regions of this gene. Introducing the aspect of dietary patterns, another study conducted in rats revealed epigenetic changes in hypothalamic appetite-regulating genes associated with the development of obesity in adult rats on a high-carbohydrate diet (82).

While our study has not established a causal link between *NR3C1* gene methylation and adherence to an industrialized diet, it underscores the need for future outcome-focused research and longitudinal studies with molecular collections. Such investigations can contribute to a clearer understanding of the mechanisms underlying this relationship. Encouraging not only nutritional but also educational and economic interventions is crucial in the promotion of healthy patterns of consumption and in reducing adherence to industrialized diets. Ultimately, these efforts may obstruct the onset of diseases through epigenetic mechanisms.

The industrialized dietary pattern has been gaining in adherence over recent years due to the movement known as nutritional transition. This trend can be extended to national and international levels; given the molecular results presented here, this indicates that we may observe a risk in terms of the development of diseases not only in the case of the study population but also among other populations. The present study suggests that industrialized (processed and ultra-processed) food can impose metabolic stress on the *NR3C1* gene, leading to increased DNA methylation in CpGs 44–47 of the IF region, a region affected by early stress and, as we were able to see here, by current metabolic stress.

## 5 Conclusion

In conclusion, we have described a positive relationship between the intake of “processed” foods and methylation levels of CpGs of component 1 (44–47) of the *NR3C1* gene, which also showed a relationship with metabolic alterations, such as alterations in glucose, cortisol, and HDL and LDL cholesterol levels, TyG index, and BMI. These findings allow us to suggest that food can be regarded as a stressor agent capable of altering the epigenetics of the stress response, taking into account socioeconomic, psychosocial, and metabolic confounding factors. In addition, this can contribute to the etiology of several diseases, including depression, metabolic syndrome, and obesity.

Although our study has not demonstrated a causal relationship, the findings suggest that industrialized food (processed and ultra-processed) can impose metabolic stress on the *NR3C1* gene, leading to increased DNA methylation in CpGs 44–47 of the IF region, a region affected by early stress and, as we have observed, by current metabolic stress.

Therefore, these results show the importance of reducing adherence to industrialized dietary patterns, ultimately thwarting the onset of disease through epigenetic mechanisms.

## Data availability statement

The original contributions presented in the study are available at https://drive.google.com/drive/folders/133MZj4n6uITw-yCfnrC5ZJo7RW0QZDCu?usp=sharing.

## Ethics statement

The studies involving humans were approved by the Ethics and Research Committee of the Health Science Center of the Federal University of Espirito Santo (Ethics statement is Number: 1.574.160 – CAAE: 52830216.5.0000.5060 obtained on the 6th June 2016, and substantiated opinion number: 3.420.734 – CAAE:08454919.5.0000.8151 obtained on the 27th June 2019). The studies were conducted in accordance with the local legislation and institutional requirements. The participants provided their written informed consent to participate in this study.

## Author contributions

TV contributed to the writing of the article, conducted data analysis, and participated in discussions. FF was involved in data analysis and discussion of the main findings. LS prepared the figures and contributed to discussion of the data. AB reviewed the manuscript. SM participated in the analysis and interpretation of food consumption data. AO reviewed the manuscript. IM participated in writing the manuscript. BQ contributed to the molecular analysis and data analysis. MS also participated in writing the manuscript. WB participated in the collection of molecular and biochemical data. JAr was involved in the collection of molecular and biochemical data. BS conducted the molecular analyses. JAs was involved in the collection of molecular and biochemical data. AA contributed to the collection of biochemical data. JS performed DNA extractions. LA participated in the discussion of molecular results following pyrosequencing analysis. DO contributed to data analysis and discussion of the main findings. AS supervised the project and guided the writing of the manuscript. All authors contributed to the article and approved the submitted version.
