# Supplementary material for: An industrialized diet as a determinant of methylation in the 1F region of the NR3C1 gene promoter
Source: Front Nutr. 2024 Apr 3;11:1168715. doi: 10.3389/fnut.2024.1168715 (PMC11021719; doi:10.3389/fnut.2024.1168715)
Supplement: Supplementary file 1 [file Data_Sheet_1.PDF]

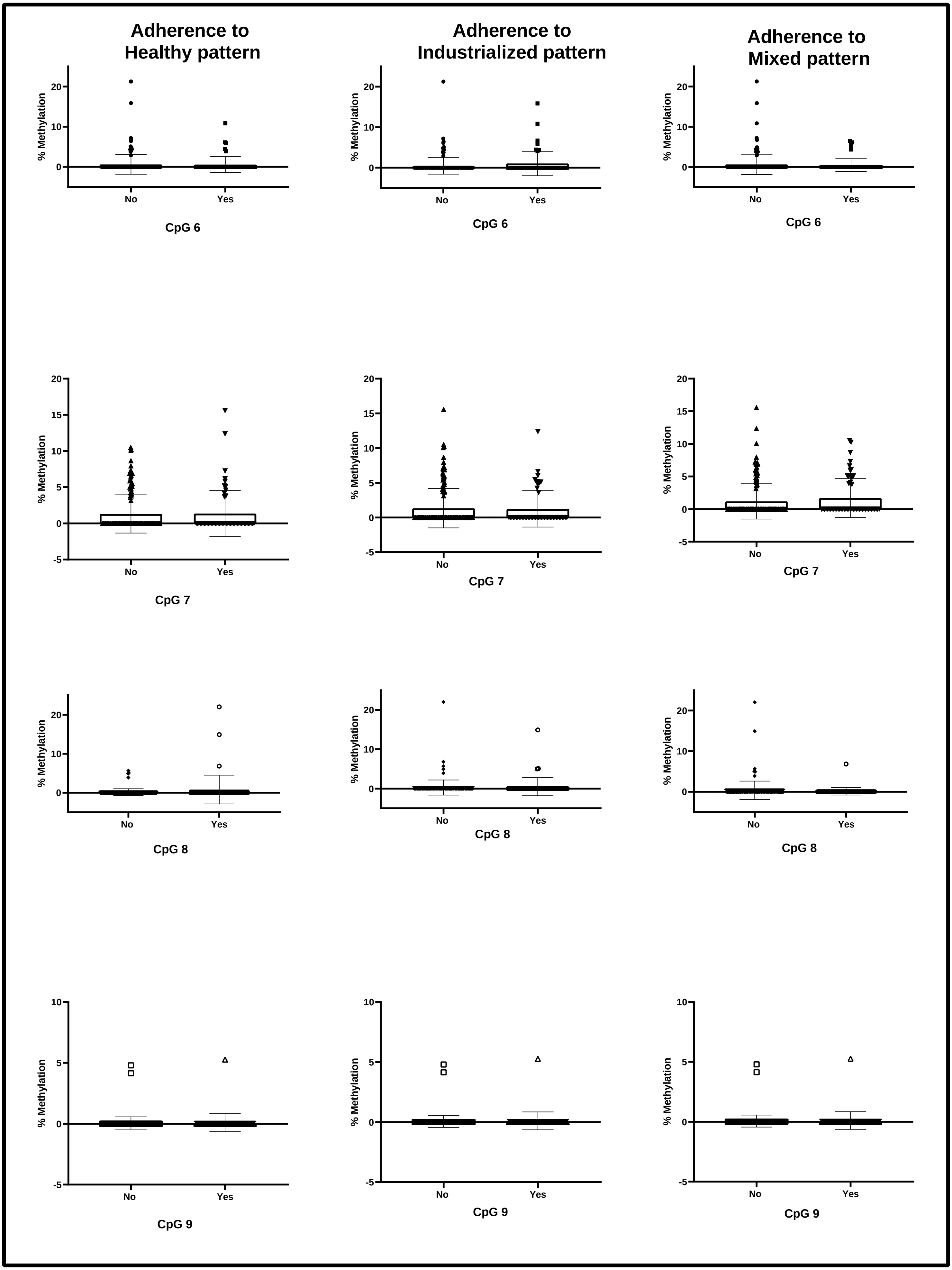

Adherence to Healthy pattern
Adherence to Industrialized pattern
Adherence to Mixed pattern
% Methylation
No
Yes
CpG 6
CpG 7
CpG 8
CpG 9
