# Supplementary figures and images for: An industrialized diet as a determinant of methylation in the 1F region of the NR3C1 gene promoter
Source: Front Nutr. 2024 Apr 3;11:1168715. doi: 10.3389/fnut.2024.1168715 (PMC11021719; doi:10.3389/fnut.2024.1168715)

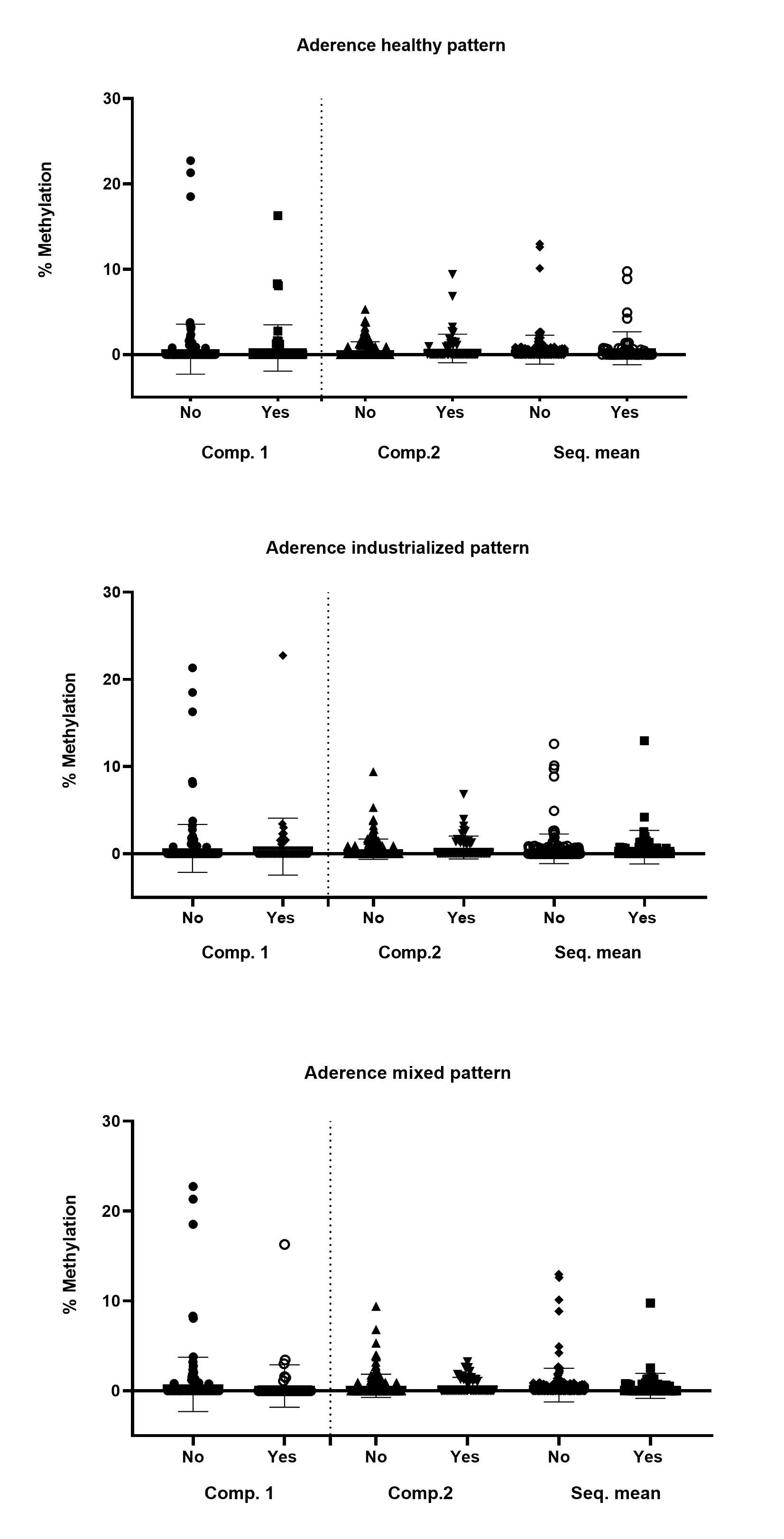

Supplement: Supplementary file 3 [file Image_1.JPEG]

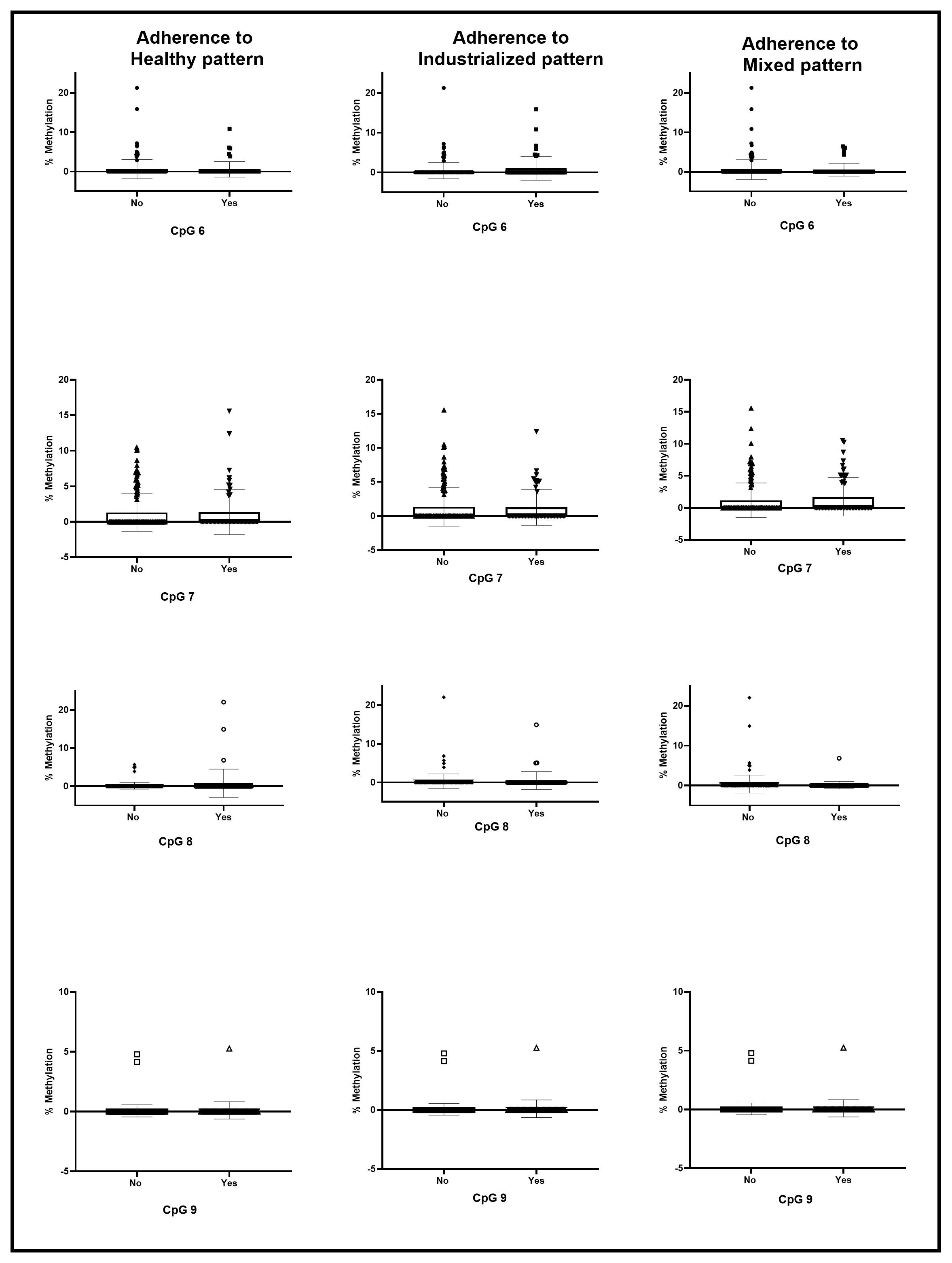

Supplement: Supplementary file 4 [file Image_2.JPEG]

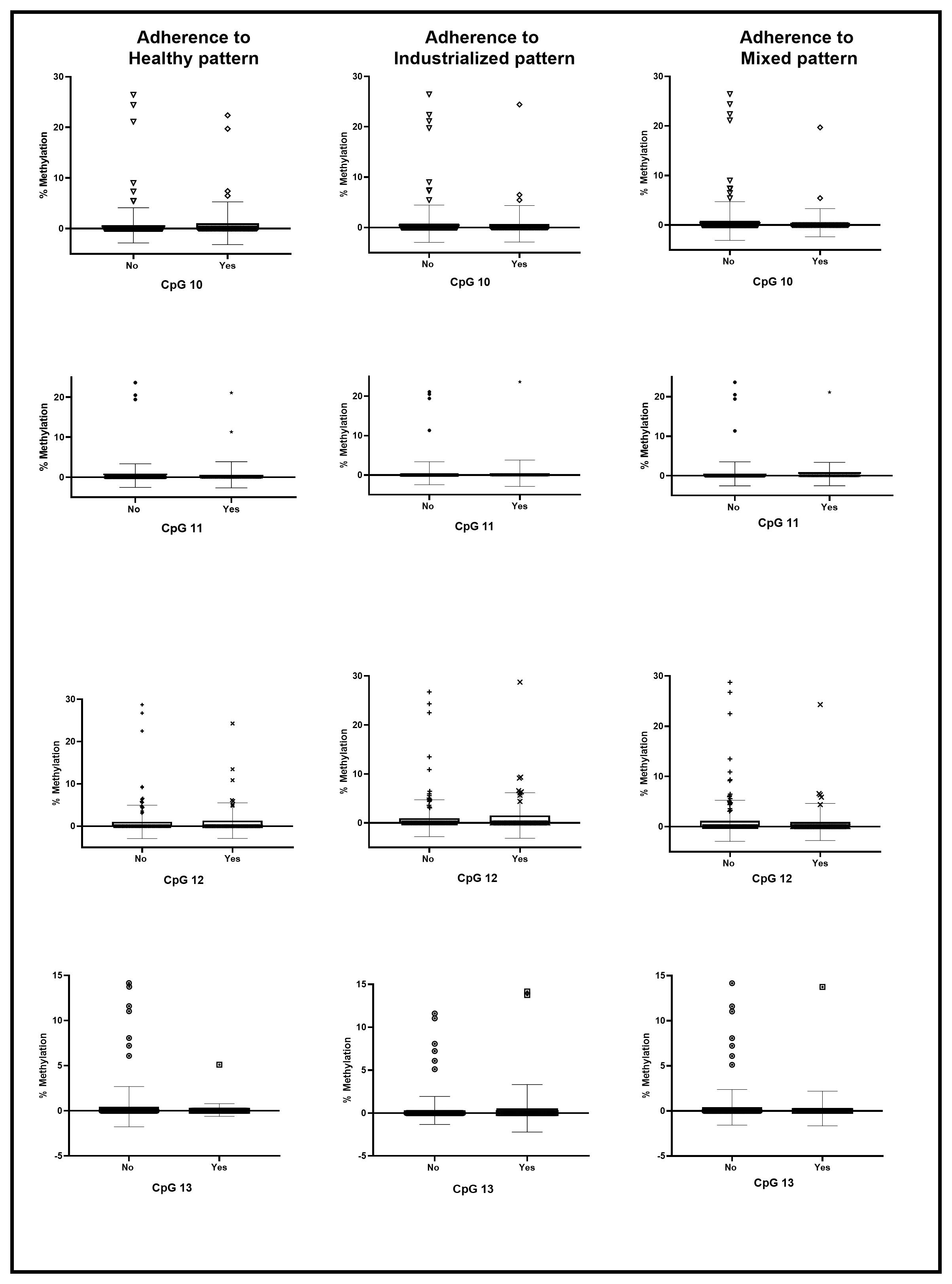

Supplement: Supplementary file 5 [file Image_3.JPEG]
